# Microbiota, Oxidative Stress, and Skin Cancer: An Unexpected Triangle

**DOI:** 10.3390/antiox12030546

**Published:** 2023-02-21

**Authors:** Barbara Azzimonti, Chiara Ballacchino, Paola Zanetta, Marie Angele Cucci, Chiara Monge, Margherita Grattarola, Chiara Dianzani, Giuseppina Barrera, Stefania Pizzimenti

**Affiliations:** 1 Laboratory of Applied Microbiology, Department of Health Sciences (DiSS), Center for Translational Research on Allergic and Autoimmune Diseases (CAAD), School of Medicine, Università del Piemonte Orientale (UPO), Corso Trieste 15/A, 28100 Novara, Italy; 2 Department of Clinical and Biological Science, University of Turin, 10125 Turin, Italy; 3 Department of Scienza e Tecnologia del Farmaco, University of Turin, 10125 Turin, Italy

**Keywords:** microbiota, oxidative stress, skin cancer, melanoma, keratinocyte carcinomas, basal cell carcinoma, squamous cell carcinoma

## Abstract

Mounting evidence indicates that the microbiota, the unique combination of micro-organisms residing in a specific environment, plays an essential role in the development of a wide range of human diseases, including skin cancer. Moreover, a persistent imbalance of microbial community, named dysbiosis, can also be associated with oxidative stress, a well-known emerging force involved in the pathogenesis of several human diseases, including cutaneous malignancies. Although their interplay has been somewhat suggested, the connection between microbiota, oxidative stress, and skin cancer is a largely unexplored field. In the present review, we discuss the current knowledge on these topics, suggesting potential therapeutic strategies.

## 1. Introduction

The incidence of both non-melanoma-skin cancers (NMSCs), and melanoma skin cancers has been increasing over the past years. Globally, in 2020, 1,198,073 new NMSC cases and 63,731 deaths occurred worldwide; of them, 324,635 were new melanoma cases, which caused 57,043 deaths [1].

Recent evidence points out the role of microbiota, the unique combination of micro-organisms residing in a specific environment, in the pathogenesis of several diseases, including skin cancer. Moreover, a state of imbalance in the microbial community, named dysbiosis, can induce oxidative stress, a well-known emerging force involved in the onset of various human pathological conditions, including cutaneous malignancies. Thus, the connections between skin cancer and microbiota dysbiosis, skin cancer and oxidative stress, or microbiota dysbiosis and oxidative stress are well-documented. From this, a causative connection between microbiota dysbiosis and cutaneous malignancies can be supposed through an alteration of the redox status. Although few publications sustain this hypothesis, in this review, after a brief presentation of the most common skin cancer types, the connections between these three elements and potential therapeutic strategies are presented.

## 2. Skin Cancer

The main skin cancer types are melanomas, which arise from the malignant transformation of melanocytes, and NMSCs, such as basal (BCC) and squamous cell carcinoma (SCC), both originating from keratinocytes and their precursors. BCC and SCC are the first and the second most common skin cancer types, respectively. However, although less frequent, melanoma in the advanced stage is the most aggressive skin cancer with the worst prognosis and is responsible for the most skin cancer-related deaths.

### 2.1. Cutaneous Melanoma

Melanoma accounts for about 1% of all skin cancers diagnosed; however, it is the most aggressive. There are about 160,000/year of new cases globally, mainly distributed in North Europe among Caucasian people, and 7000/year of new epidermal cancer-associated deaths in Italy alone [2]. Its incidence is high in Australia, which holds a sad record due to excessive sun ultraviolet (UV) light exposure [3]. Nevertheless, in the last ten years, the general incidence of cutaneous melanoma has increased by about 15% compared to the previous decade worldwide [4].

In recent years, melanoma has been studied intensively in terms of the immune response and specific mutations to develop new biological therapies [5]. Surgical extirpation remains the mainstay of curative-intent treatment for malignant melanoma. When detected at an early stage, the tumor can be removed by minor or extensive local resection and lymphadenectomy [6]. In sentinel-node-positive patients, lymphadenectomy with routine ultrasonographic surveillance has been shown to limit morbidity. However, in advanced stages, the presence of metastasis comprehensive treatments, including radiotherapy and drug therapy, is indispensable [7].

Several studies have demonstrated that the spread of melanoma depends both on genetic mutations and microenvironmental tumor alterations. Melanoma driver mutations often occur in regulating proliferation, metabolism, apoptosis, and cell cycle control [8]. About 50% of melanomas harbor activating mutations of the B-Raf proto-oncogene (BRAF). More than 85% of them have a valine–glutamic acid substitution in codon 600 of the BRAF (V600E), which leads to constitutive activation of downstream mitogen-activated protein kinase (MAPK) signaling [9]. The second most common cause of aberrant activation of the MAPK pathway in cutaneous melanoma is represented by NRAS-activating mutations (15–30% of melanomas) [10]. Interestingly, BRAF-mutated melanoma cells are generally NRAS wild type, and vice versa; thus, BRAF and NRAS mutations are considered mutually exclusive at the single cell level. However, NRAS- and BRAF-activating mutations can coexist within the same melanoma specimen in different melanoma subclones [11].

In melanoma, the phosphoinositide 3-kinases (PI3K) pathway, usually involved in cellular homeostasis, is the second most frequently activated signaling [12,13]. Other mutations lead to the overexpression of proteins able to favor tumor invasion and surrounding infiltration, such as endothelin1 [14], and metalloproteinases (MMPs) [15]; among the latter, MMP2 [16] and MMP9 [17], which are regulated by the inhibition of nuclear factor-κB (NF-κB) signaling pathway [18,19], are particularly relevant.

Cell adhesion molecules also play a role in melanoma cell migration and metastasis. Indeed, in early-stage melanomas, there is a loss of E (epithelial)-cadherin expression, which is associated with a loss of communication with the regulatory keratinocytes in the epidermis, and a shift to N (neuronal)-cadherin expression that allows melanoma cells to preferentially bind to fibroblast cells and thus promote invasion into the dermis [20].

Melanoma mutations may also involve neo-antigens, which are degradation products of proteins presented by the major histocompatibility complex (MHC) and can be the target of cancer immunotherapy [21]. The upregulation of the MAPK pathway, as observed in BRAF-mutated melanoma cells, can induce immune-escape mechanisms that make melanoma cells able to evade T-cell immune responses [22].

Unfavorable immunologic features are reversed by therapy with BRAF inhibitors (vemurafenib, dabrafenib, and encorafenib) and mitogen-activated protein kinase kinase (MEK) inhibitors (cobimetinib and trametinib) [23,24].

The stimulation of the immunological response in melanoma was also obtained by the inhibition of Cytotoxic T-Lymphocyte Antigen 4 (CTLA-4), an immunoglobulin cell surface receptor, able to inhibit T-cell activation [25,26]. In 2011, the Food and Drug Administration (FDA) approved the use of ipilimumab, a fully human, IgG1κ monoclonal, anti-CTLA-4 antibody in advanced melanoma. Another attempt to improve the immunological response in advanced melanoma has been made by targeting Programmed Cell Death Protein 1 (PD-1). PD-1 is an immune checkpoint with a central role in immunopathology and tumor immune surveillance through effector T-cell inhibition [27]. In 2014, the FDA approved two monoclonal antibodies targeting PD-1 (nivolumab and pembrolizumab) for the treatment of metastatic melanoma. Randomized clinical trials showed that monotherapy with nivolumab or pembrolizumab is superior to ipilimumab alone [28]. Moreover, therapy concurrently targeting CTLA-4 and PD-1 may confer enhanced clinical outcomes compared to monotherapy [29].

Oncogene inhibitors and immune checkpoint inhibitors have revolutionized the treatment of patients with advanced-stage metastatic melanoma; however, a subset of patients who initially respond to therapy later relapse and develop resistance (termed “acquired resistance”), whereas others do not respond at all (termed “primary resistance”). Several studies have been performed in these years to understand the mechanisms involved in melanoma progression and resistance to therapies. Most often, resistance to targeted therapies is due to either the reactivation of the MAPK pathway or the activation of alternative kinase signaling pathways [30], while immunotherapy resistance seems to depend on epigenetic mechanisms and low tumor mutational burden, a numeric index that expresses the number of mutations harbored by tumor cells or altered immune signaling [31].

### 2.2. Keratinocyte Carcinomas

Keratinocyte carcinomas (KCs) are the most common malignancies worldwide, comprised under the vast term of NMSC. KCs include BCC, SCC, and Bowen’s Disease (BD), a superficial SCC in situ [32].

BCC is the most frequent skin malignancy, representing about 78–80% of cutaneous cancer cases [33]. It arises from basal cells, located in the deepest part of the epidermis, which have been recently considered to be skin stem cells. BCC is defined as a slow-growing tumor, which rarely metastasizes (less than 0.1%) but it can cause facial deformities if untreated [34]. A characteristic feature of BCC, which mainly develops within hair follicles, is the formation of islands or nests of basaloid cells found in the epidermis, which can invade the dermis depending on the BCC variant [34,35,36].

SCC originates from epidermal keratinocytes or adnexal structures (such as eccrine glands or pilosebaceous units) [37] and contributes to approximately 20% of skin cancer cases [38]. It is histologically characterized by the exponential growth of abnormal keratinocytes, which mainly occurs in the lower epidermis layers [39]. Actinic keratosis (AK), due to UV exposure, is the principal precursor lesion for the formation of SCC, mainly in transplant recipient immunosuppressed patients [33,40]. SCC can be divided into carcinoma in situ, also known as BD, which represents a transitional phase from AK to invasive SCC, which is often referred to as standard SCC. The atypical keratinocytes exhibit apoptosis, hyperchromasia, nuclear pleomorphism, and polarity loss [41]. Invasive SCC is characterized by an invasion of abnormal cells from the basement membrane into the dermis. The clinical variants of SCC include simplex SCC [42], acantholytic SCC [43], spindle cell SCC [39], verrucous SCC [39], clear-cell SCC [44], and single-cell infiltrates, which are often undetected or misdiagnosed [45].

Together with SCC, BCC incidence is increasing worldwide, representing about 95% of the total NMSC, with a generally good prognosis, especially if recognized early [33,46]. Despite skin cancer pathogenesis is multifactorial, UV radiations, in particular UVB, are historically recognized to be one of the main risk factors for skin carcinogenesis [47]. KC risk includes not only long-term UV exposure but also short periods of intense sun exposure or burning, especially in childhood or with sunbed use. Cumulative UV exposure over decades appears to be the main risk factor for SCC, whereas intermittent UV exposure in childhood and adolescence is the leading risk factor for BCC [48]. Moreover, UV exposure can alter the skin microbiota, leading to the massive formation of reactive oxygen species (ROS), apoptosis, and inflammation [49].

Other environmental factors involved in KC risk are ionizing radiation owing to radiotherapy, X-rays, total body irradiation and atomic bombs, and immunosuppression, due to organ transplantation, chronic leukemias and lymphomas, and human immunodeficiency virus (HIV) infection [50,51]. The role of chronic inflammation as a risk for KC was sustained by Tang and Wang [52] who identified the ROS and nitrogen species (RNS) as responsible for DNA damage and genomic instability.

Beyond the environmental factors that drive keratinocytes toward the two skin tumors, genetic factors play an important role in KC susceptibility [53]. Family histories of skin cancer were associated with a four-fold higher risk of SCC after adjustment for known environmental SCC risk factors. In models including skin cancer type, the highest association was for a family history of BCC and for multiple skin cancer types [54]. The identification of genomic susceptibility loci [55,56] supports the role of lighter pigmentation and its interaction with UV radiation exposure in the risk of KC.

Several types of treatment are available for KCs. Surgical excision is commonly used since it allows histologic examination for tumor-free margins. Electrodesiccation and curettage are used for low-risk primary, non-fibrotic tumors [57]. Topical chemotherapy with fluorouracil has also been used to treat BCC and SCC in situ [58]. Topical imiquimod (Aldara), an immunomodulator, is approved by the FDA for treating superficial BCC, but not for other BCC subtypes [59]. Moreover, immunotherapy with immune checkpoint inhibitors has recently become a successful option for treating advanced SSC [60].

## 3. Microbiota and Skin Cancer

### 3.1. Human Microbiota and Cutaneous Melanoma

Recently, the skin and gut microbiota has been suggested to play a role in cutaneous melanoma [61,62], even if the specific research in this field is still in its infancy.

Recent analysis, conducted on acral melanoma patients, reported an increased association between the *Corynebacterium* genus, found by microbial culturomic analysis of lesional skin swabs, and disease severity [63]. Moreover, a relationship was observed between the of *Corynebacterium* levels and interleukin (IL)-17 [63]. In fact, this cytokine can promote melanoma cell proliferation by increasing both IL-6 and the signal transducer and activator of transcription 3 (STAT-3) [64]. Another study reinforced these data, suggesting that specific *Corynebacterium* species stimulate the infiltration of IL-17A-producing T-cells on the dermal skin of a mouse study model [65]. 

Conversely, *Cutibacterium acnes* (formerly *Propionibacterium acnes*) activity is the opposite, since it has been demonstrated that its intra-tumoral injection in mouse models significantly decreased mouse melanoma growth and size, via the production of IL-2, tumor necrosis factor-α (TNF-α), and interferon-γ (IFN-γ) [66]. Similarly, Wang and collaborators demonstrated that bacteria supernatant from *C. acnes* increased apoptosis in UVB-irradiated melanocytes [67].

*Staphylococcus epidermidis* seems to play a role, even if controversial, that needs to be elucidated. Indeed, intravenous injection of *S. epidermidis*-derived 6-*N*-hydroxyaminopurine (6-HAP) inhibited melanoma growth in mice [68], while lipoteichoic acid (LTA), a by-product from the same bacteria, protected melanocytes survival from in vitro UVB radiation by upregulating TNF Receptor Associated Factor 1 (TRAF1), Critical Assessment of Structure Prediction 14 and 15 (CASP14 and CASP5), and Tumor Protein 73 (TP73) [67].

In a melanoma minipig study model, skin microbiome analysis reported significant differences in microbial composition and diversity between melanoma and normal skin samples [61]. In this regard, *Fusobacterium* and *Trueperella* levels were higher in melanoma samples and related to unfortunate disease progression [61], as observed for other tumor types, such as oral and colorectal cancers (CRC) [69,70]. *F. nucleatum* action is mediated by the natural killer (NK) cell activity downregulation through bacterial fatty-acid-binding protein 2 (Fap2) protein and NK receptor “T-cell immunoreceptor” interaction with immunoglobulin and immunoreceptor tyrosine-based inhibitory motif domain (TIGIT) [71].

As a matter of fact, what has been observed for CRC mucosa, in which tumoral samples are more strongly colonized by the so-called “cancer-adherent/associated microbiota” with respect to the healthy ones, could also be supposed for skin cancer [72]. Indeed, the anaerobic *F. nucleatum*, which intervenes in important biofilm-organizing habits through the Fap2 adhesin that binds to CRC D-galactose-beta [1-3]-*N*-acetyl-D-galactosamine (Gal-GalNAc) overexpressing cells, invades and creates intracellular colonies [73], where it secretes cytokines that can stimulate TNF, thus increasing the inflammatory, cancerization and metastasizing processes.

Recent evidence underlies that the onset and progression of skin cancers can be influenced not only by the skin/gut bacteriota, but also by the virota, with its oncolytic and non-oncolytic viruses [74,75]. Indeed, while the role of specific bacteria in skin melanoma has been demonstrated, the role of some viruses, such as Human Papilloma Viruses (HPVs), is still debated, since these human commensals, well-recognized to specifically and selectively target cutaneous and mucosal basal epithelial cells, do not infect melanocytes. Nevertheless, Dréau and colleagues found them in 58% of stage III and IV melanoma patient skin lesions, thus suggesting a possible role for HPV-positive melanocytes in promoting more aggressive tumor behavior [76]. However, a retrospective analysis of formalin-fixed paraffin-embedded melanoma biopsies did not evidence any statistical correlation between HPV DNA presence, observed via molecular Polymerase Chain Reaction (PCR) and Restriction Fragment Length Polymorphism (RFLP) assays, and the patient’s clinical outcome [77]. Conversely, another study [78] revealed that mucosal HR-HPV DNA and Human Melanoma Black-45 (HMB-45), a tumoral melanocytic marker, strongly co-localized in primary melanomas, thus reinforcing its active part in this tumor type. Other analyses addressed this theme; in a population-based cohort study, an association between HPV infection and increased melanoma risk was retrieved, as demonstrated by high-risk mucosal HPV16 and 33 genotypes in 27% of MM skin biopsy detection [75,79]. Regarding other mucosal HPV types, their active role was demonstrated in uveal melanomas: by downregulating HPV 18 E6/E7 expression, the tumor growth was inhibited, and the cell cycle was blocked through p53 and pRb pathway activation [80]. Conversely, among β-HPVs genotypes, HPV22 resulted more frequently in injured skin with respect to the same individual’s healthy skin, even if the patient’s clinicopathological features were not specifically linked with HPV prevalence [81].

Regarding Merkel Cell Polyomavirus (MCPyV) and its possible association with melanoma incidence, the PCR-based research of Koburger and collaborators on 95 paraffin-embedded primary melanoma samples did not reveal any association [82]. Furthermore, Mokanszki et al. [83] identified four MCPyV-positive samples in 60 cutaneous melanomas, finding only a little association between the infection and melanoma severity. Up to now, the pathogenic relationship between MCPyV and melanoma still needs to be further clarified.

With respect to human endogenous retrovirus (HERV), Singh and colleagues found virus-like particles, mRNA, proteins, and antibodies in melanoma patients. Since these viruses are ubiquitous within the population, it is not so obvious that they can have a role in melanoma pathogenesis [84]. Nevertheless, upon UVB irradiation, the expression of specific viral genes, such as those coding for the envelope and polymerase, resulted in being associated with melanocyte transformation and immune evasion [84].

Not only the skin but also gut microbiota has gained a major role in melanoma progression and resistance to therapies. Thanks to the sequencing of bacterial 16S rRNA and the fungal internal transcribed spacer region on fecal samples of early- and late-stage melanoma patients and healthy people, Vitali and colleagues correlated the microbial profiles with the histopathological features of melanoma [62]. After a complete microbial meta-analysis, they found peculiar gut microbiota and bacterial fingerprints which differentiate patients from those who are healthy. Melanoma-affected patients showed high *Prevotella copri* and *Saccharomycetales* loads. Interestingly, those bacterial and fungal communities were related to the lesion invasiveness, while specific fungi to its regression. Moreover, the bacterial diversity of metastatic melanoma patients was reduced with respect to the phase I and II ones and correlated with tumor features, worse disease progression and therapy response [62]. Moreover, gut microbiota composition has been recognized as one of the novel biomarkers able to accurately predict the subset of patients who would benefit from immunotherapy with CTLA-4 and PD-1 blockers. In particular, fecal samples enriched in *Firmicutes* phylum were associated with a good response to immunotherapy; conversely, the *Bacteroidales* family was associated with a poor response to immunotherapy [85]. Thus, the modulation of gut microbiota through the control of dietary habits, the administration of prebiotics or probiotics, or even Fecal Microbiota Transplantation, has become an attractive approach for treating advanced melanoma [86].

### 3.2. Microbiota and Keratinocyte Skin Cancer

To date, the most recent research focuses on the role of the microbiota in KCs [87], but little is known about its composition, mediators, and role in the genesis, progression, and response to therapy. The biological barrier, represented by the skin microbiota, inhibits pathobiont and pathogen invasion through the secretion of antimicrobial peptides (AMPs) such as cathelicidin LL-37 and human β-defensin, thus creating crosstalk with keratinocytes and immune cells [88,89,90]. In addition, microbes metabolize some lipids of the skin surface, such as sapienic acid, which has antimicrobial activity, and triglycerides, which can be bioactive against other micro-organisms or stimulate host cell action [91].

When the skin barrier is disrupted or a condition of dysbiosis occurs, local skin or systemic infection-related diseases are a possible consequence [92]. As an example, while strong evidence associates bacteria prevalence like *S. aureus* with AK severity, leading one to think that inflamed lesioned skin accelerates bacterium colonization and vice versa, the association with SCC is not yet fully understood [40]. In this regard, in a case-control study, Kullander and collaborators compared *S. aureus* occurrence in tumoral skin lesions and swabs from patients with different cutaneous lesioned and non-lesioned skin [93]. These authors did not find any association between *S. aureus* and BCC/seborrheic keratosis, but they found a significative higher load of this bacterium in AK, and even more in SCC with respect to healthy skin samples [93]. This is of particular interest since AK can progress to SCC. Moreover, since *S. aureus* is the most represented bacterial skin species, these authors sought to understand whether its high concentration was due to the increased susceptibility of SCC-affected skin or whether the bacterium involvement was casual. They observed that, although *S. aureus* is a skin microbiota commensal, this bacterium is not able to infect the skin of immunocompetent subjects unless they present epithelial lesions; thus, they suggested that the association between *S. aureus* and SCC is not casual, and that the skin ulceration is a result of the pathogenetic process that favors exogenous prevarication/infection [93]. Moreover, *S. aureus* could also participate in the pathogenesis of SCC, causing chronic local inflammation, which is involved in different tumorigenic stages including (i) survival promotion, (ii) proliferation, (iii) cell transformation, (iv) invasiveness, (v) angiogenesis, and (vi) metastatization [94]. Further confirmation of the pathogenic role of *S. aureus* in SCC onset was recently obtained by Voigt and collaborators [95]. These authors demonstrated that the relative abundance of this pathobiont was increased at the expense of commensal *C. acnes* in SCC compared with that in healthy skin.

Several human neoplasms are favored by infections promoted by opportunistic or primary pathogens. The main mechanisms by which dysbiotic patterns and exogenous or endogenous infections favor cancer development are the insertion of oncogenes and/or the inhibition of immune cells to counteract neoplastic growth [93]. One of the main mediators is the TNF-α, whose upregulation promotes neo-angiogenesis and tumor progression. Recent studies have shown that staphylococcal toxin-α determines its secretion by local cells involved in the inflammatory process, which, in turn, leads to the activation of nuclear factor kappa-light-chain-enhancer of activated B cells (NF-Kβ), with a consequent increase in the expression of different cytokines and chemokines, including IL-1β, IL-6, and IL-12 [93,96]. A further factor that mediates skin inflammation from *S. aureus* is the virulence peptide modulin phenol-soluble-α (PSMα). PSMα induces damaged keratinocytes to release IL-1α and IL-36α, which act (i) with an autocrine mechanism on IL-1R and IL-36R, and (ii) by means of the Myd88 adapter at the level of Tγδ lymphocytes, promoting the secretion of IL-17, a cytokine essential in mediating the inflammatory response against *S. aureus* [97,98].

The link between microbial dysbiosis, chronic inflammation, immune evasion and oxidative stress has already been reported for *Helicobacter pylori* in gastric tumors and for *F. nucleatum* in CRC [99,100]. Despite the bond between skin microbiota, ROS and NMSC occurrence is still largely unknown, the state of dysbiosis, which causes a greater susceptibility to exogenous and non-exogenous stimuli, also favors DNA damage, such as the formation of thymine dimers and C-T transitions, erythema, immunosuppression, melanogenesis, photo-ageing and cancer [101,102].

## 4. Microbiota and Oxidative Stress

### 4.1. Oxidative Stress in the Skin

The balance between oxidants and antioxidants, in favor of the oxidants, is defined as oxidative stress. This condition is characterized by an excess production of ROS or RNS relative to antioxidant defenses. 

ROS/RNS are continuously produced in living organisms. They include free radicals, such as superoxide anion (O_2_^•−^), hydroxyl radical (•OH), and nitric oxide (NO^•^), as well as non-radical species such as singlet oxygen (^1^O_2_), peroxides (hydrogen peroxide H_2_O_2_; peroxynitrite ONOO^−^), and hypochlorous acid (HOCl) [103]. Under oxidative stress, the excess of ROS/RNS can damage nucleic acids, proteins, and lipids. 8-hydroxy-2′-deoxyguanosine (8-OHdG) is one of the most abundant oxidative products of DNA; proteins can be damaged by the oxidation of amino acid residue side chains; finally, ROS/RNS trigger polyunsaturated fatty acid (PUFA) oxidative degradations in lipid membranes. This process is known as lipid peroxidation and induces the formation of lipid radicals (lipid radical L^•^, lipid peroxy radical L-OO^•^, and lipid hydroperoxide L-OOH), and other reactive molecules such as 4-hydroxynonenal (HNE) and malondialdehyde (MDA), able to further amplify the toxic effect of free radicals [104].

A large variety of antioxidants can protect molecules from oxidative stress-related injury. They include enzymes, such as superoxide dismutases (SODs), glutathione peroxidases (GPXs), peroxiredoxins (PRDXs), catalase (CAT), and non-enzymatic molecules such as glutathione (GSH), Vitamin A, C, and E [105]. The master regulator of the cytoprotective and antioxidant response is the transcription factor NF-E2-related factor 2 (Nrf2). Under oxidative stress, it activates the transcription of several antioxidant genes (i.e., PRDXs) including those involved in GSH synthesis [106].

ROS/RNS can be locally produced in skin cells by several mechanisms. As in other mammalian cells, ROS such as O_2_^•−^ and ^1^O_2_, are generated in mitochondria during oxidative phosphorylation; O_2_^•−^ is synthesized by NADPH oxidase (NOX) and xanthine oxidase (XO); the free radical NO^•^ is one of the RNS generated by NO synthase (NOS). Moreover, with respect to other tissues, ROS/RNS skin can be further generated by the exposition to several environmental factors, such as UV radiations (UVA and UVB), pollution, including cigarette smoke, and particulate matter (PM) [107]. Together with various genetic or non-genetic internal factors, these components define the skin exposome as the sum of every exposure to which an individual is subjected from conception to death [108]. UV irradiation, the leading cause of skin cancer, is one of the main initiators of ROS generation in the skin. Besides direct DNA damage [109], UV rays induce the skin to produce ROS through the involvement of photosensitizer molecules such as riboflavin, cytochromes, heme, and porphyrin [110]. These excited photosensitizer molecules can then react with oxygen, resulting in the generation of ROS (O_2_^•−^ and 1^1^O_2_); SOD can transform O_2_^•−^ to H_2_O_2_, which, in turn, thanks to Fe (II) or Cu (III), lead to the production of •OH (Fenton reaction) [111]. Moreover, UV can directly activate NOXs, with a consequent ROS production [112]. PM originating from fuel combustion can contain several molecules able to enhance ROS production. For instance, polycyclic aromatic hydrocarbons (PAH) contained in the PM, besides their carcinogenic role, are also photosensitizer molecules; thus, they can induce ROS production under UV exposure [113]. Ryu and collaborators have demonstrated that PM with a diameter of ≤2.5 (PM2.5) stimulates skin keratinocytes to produce various inflammatory cytokines, and ROS [114].

Another major player in the mechanism of ROS production is the aryl hydrocarbon receptor (AhR) [115]. This ligand-activated transcription factor can control the expression of several genes in a cell-type-specific and ligand-specific manner. At first, its ability to act as a sensor of xenobiotic chemicals such as aromatic (aryl) hydrocarbons was discovered, from which the receptor derives its name. Upon ligand binding, AhR can translocate into the nucleus, where it dimerizes with AhR nuclear transporter (ARNT) or other partners; then, it binds to the xenobiotic-responsive element (XRE) and induces the transcription of AhR-responsive genes, such as the xenobiotic-metabolizing enzymes belonging to the cytochrome p450 family (CYP1A1, CYP1A2, and CYP1B1), also able to detoxify these chemicals. The components of air pollution, such as PAH or PM2.5, some food molecules (e.g., polyphenols), but also some endogenous amino acid derivatives, such as 6-formylindolo[3,2-b]carbazole (FICZ), the photoproduct of the UV irradiation of L-tryptophan, can bind AhR [115]. During these enzymatic reactions, a large amount of ROS is produced so that the activation of AhR elicits the induction of oxidative stress, cytokine expression and DNA damage [116,117]. It has been shown that keratinocytes exposed to Benzo(a)pyrene (BaP), an environmental contaminant found in cigarette smoke, induce an Ahr-dependent production of ROS and IL-8; this mechanism can explain, at least in part, how cigarette smoke can worsen some inflammatory skin diseases such as psoriasis or acne [118]. Moreover, AhR, activated after PM exposure, induced ROS through the involvement of NADPH oxidase, which in turn can activate both NF-κB and activator protein 1 (AP-1), leading to an induction of COX-2 and subsequent increase in the pro-inflammatory PGE2 [119].

Interestingly, in skin melanocytes, O_2_^•−^ and H_2_O_2_ can also be produced during melanin synthesis, although the confinement of this synthetic pathway to melanosomes protects other cellular components from oxidative damage [120].

ROS can exacerbate inflammatory skin diseases. For instance, they play a crucial role in the pathogenesis of atopic dermatitis (AD), psoriasis, and vitiligo [121].

In the serum or urine of AD patients, oxidative stress biomarkers, such as MDA, lipid LOOHs, NO^•^, and 8-OHdG, have been detected [122,123,124,125]; in addition, antioxidants, such as SOD, CAT, GSH, GPX, vitamins A, C, and E, were lower in AD patients compared to controls [126,127]. A high level of oxidative stress is associated with the worst prognosis of AD diseases. In the serum of psoriasis patients, oxidative stress biomarkers, such as MDA and NO^•^, were found to increase, while SOD and total antioxidant capacity (TAC) were reduced [128].

An increased ROS level was found in the keratinocytes, fibroblasts, and neutrophils of the skin in psoriasis patients [129]. Moreover, NOX4, which is expressed in dermal fibroblasts, was found to be indispensable for keratinocyte proliferation [130], and ROS was involved in neutrophil chemotaxis [131].

In vitiligo patients, Li and collaborators found significant increases in the levels of MDA and 8-OHdG, while CAT, SOD, and TAC were downregulated; moreover, high levels of oxidative stress correlated with the worst prognosis [132]. Moreover, high ROS levels in the skin cause melanocyte destruction, leading to the depigmentation area characteristic of this disease [133].

Inflammation and oxidative stress are intimately connected and mutually reinforcing. On the one hand, many of the molecules able to induce oxidative stress are produced by infiltrated inflammatory cells. In fact, activated neutrophils and monocytes can increase the production of H_2_O_2_, •OH, O_2_^•−^, and ONOO^−^ [134]. Moreover, ROS/RNS and reactive sulfur species (RSS), such as hydrogen sulfide (H_2_S), can stimulate cell metabolism, and the worsening of the inflammatory status [135]. They can also influence several signaling pathways, such as the NF-κB pathway, which can induce the expression of various pro-inflammatory genes [136]. For instance, in AD patients, oxidative stress, implicated in the pathogenesis of this disease [137], activates the NF-kB pathway, with the subsequent induction of many inflammatory cytokines (such as IL-6, IL-8, IL-9, and IL-33) and the worsening of skin inflammation [138].

### 4.2. Regulation of Redox Skin Level from Microbiota

Human skin bacteria can protect from endogenous and exogenous pathogens by saturating the free adhesion sites and producing antimicrobial peptides. Conversely, when a dysbiosis occurs, resilient pathobionts not only survive but also proliferate in excess, inducing oxidative stress via the epithelial production of ROS species, with highly conserved mechanisms among the bacterial phyla [108,139], with an increase in the oxidative stress level. For instance, *C. acnes* can stimulate keratinocytes to produce ROS through the cytosolic NAD(P)H oxidase [140]; moreover, ROS/RNS and, in particular, NO^•^, are directly produced by keratinocytes in response to *S. aureus* metabolite exposure [141].

Several environmental factors of the skin exposome, such as UV rays or PAH, are well-known inducers of both cutaneous dysbiosis and oxidative stress, as reported in the previous paragraph. However, while the mechanisms of ROS production after UV and PAH chronic exposure in the skin are well clarified, the contribution of cutaneous microbiota in the generation of oxidative stress in these conditions has not yet been completely elucidated. In this regard, an important role seems to be played by AhR. This chemical sensor not only can bind several components of the skin exposome (i.e., PAH, PM), as reported above, but it can also bind bacterium or yeast metabolites, leading to the induction of ROS production and inflammation. *Malassezia* yeasts, commensal cutaneous micro-organisms, are implicated in the pathogenesis of some dermatological inflammatory diseases such as pityriasis versicolor (PV), seborrheic dermatitis (SD), or AD; interestingly, skin extracts from patients with these *Malassezia*-associated diseases demonstrated a 10–1000-fold higher AhR-activating ability than control skin extracts. The yeast products Indirubin, 6-formylindolo[3,2-b]carbazole (FICZ), and indolo[3,2-b]carbazole (ICZ) have shown the highest capacity to induce AhR [142]. Of note, AhR activation by microbiota metabolites does not always lead to the induction of oxidative stress and inflammation. For instance, tryptophan (Trp) metabolites produced by bacteria, in particular Indole-3-aldehyde (IAId), bind AhR, but they negatively regulate skin inflammation in AD patients [143]. Moreover, commensal cutaneous microbes, through the activation of AhR signaling, have been found necessary for normal epidermal differentiation, epidermal permeability barrier (EPB) function, and repair [144]. These findings underline the dual role of AhR [145] with both pro- or anti-inflammatory roles [146], as well as with pro or antioxidant functions [117].

Conversely, probiotics have been known for many beneficial health effects, including their antioxidant activity, and the ability to prevent and revert the toxic effects elicited by ROS. The antioxidant activity of probiotics is supported by eight major mechanisms, as proposed by Wang and collaborators [147]: (1) probiotics, such as *Streptococcus thermophilus* 821 or *Lactobacillus casei* KCTC 3260, showed a chelating ability for either Fe^2+^ or Cu^2+^, thus inhibiting the Fenton reaction and the production of ROS; (2) probiotics have their own antioxidant enzymes, such as SOD (*L. fermentum* E-3 and E-18) or CAT (*L. casei* BL23); (3) *Bifidobacterium* and *Lactobacillus* probiotics can produce different kinds of metabolites with antioxidant activity, such as GSH, butyrate, and folate; (4) *Lactobacillus* probiotics can increase SOD, GPx, and CAT of the host; (5) probiotics can induce the expression of the host metabolites with antioxidant activity (GSH, folate); (6) probiotics can modulate signaling pathways, i.e., activation of Nrf2, the master regulator of the antioxidant response; (7) probiotics inhibit the activity of enzymes producing ROS, such as NOX; (8) probiotics can regulate the intestinal microbiota composition, by inhibiting the excessive proliferation and virulence of pathological bacteria with potential pro-oxidant activities.

Specifically in the skin, the cutaneous microbiota can also produce several metabolites with antioxidant properties. For instance, skin microbiota, together with keratinocytes, is able to produce copious amounts of volatile organic compounds (VOCs), which include several α,β-unsaturated, saturated, or aromatic aldehydes. These compounds have been demonstrated to have a protective action towards environmental factors by activating the Nrf2-keap1 pathway [148]. In particular, it has been demonstrated that benzaldehyde, an aromatic aldehyde that is a well-known metabolite of *S. epidermidis*, is able to induce the Nrf2-keap1 pathway in human keratinocytes [148]. Moreover, these authors identified a newly described aromatic aldehyde, 3-furaldehyde (3-FA), produced by *S. epidermidis* and *S. aureus* cultures, which also activated this pathway. Interestingly, the Nrf2-keap1 induction elicited significant protection against UVB-induced keratinocyte apoptosis [148].

Lee and collaborators investigated the role of *S. epidermidis* WF2R11 in reducing the oxidative stress in HaCaT keratinocytes caused by the fine particulate matter PM2.5. This air pollutant was able to stimulate AhR to produce ROS and inflammatory cytokines, eliciting apoptosis. These authors found out that the supernatant derived specifically from the *S. epidermidis* WF2R11 was able to revert these PM2.5-induced effects by inhibiting AhR activation [149].

From the metabolites of *C. acnes*, an abundant skin commensal, the RoxP (Radical oxygenase of *C. acnes*) protein was isolated, with natural antioxidant properties. It was demonstrated that RoxP protected keratinocytes and monocytes from oxidative stress damage; moreover, this bacterium, together with the RoxP expression, exhibited a diminished prevalence in oxidative skin disease [150].

Finally, in addition to the cutaneous microbiota, it is necessary to underline the role of the gut microbiota in several dermatological diseases, including acne, psoriasis, and AD [151], which are characterized by high levels of inflammation and oxidative stress, as previously described. Indeed, several studies have shown the bidirectional relationship between gut microbiota and skin, the so-called ‘gut–skin axis’, well reviewed elsewhere [152]. Hence, both gut and skin dysbiosis is associated with an altered immune response, promoting the development of skin diseases. Gut dysbiosis elicits a dysfunctional intestinal barrier, with an increase in inflammatory mediators, pro-oxidant species, and metabolites released by the micro-organisms. Mounting evidence has also shown the potential beneficial effects of probiotics in this context, able to restore the physiological gut microbiota profile [152,153]. Indeed, probiotic administration, which is able to reduce systemic oxidative stress [154], has been successfully used to treat adult patients with skin inflammatory diseases, such as AD [155] and psoriasis [156].

## 5. Oxidative Stress and Skin Cancer

**Figure 1:**
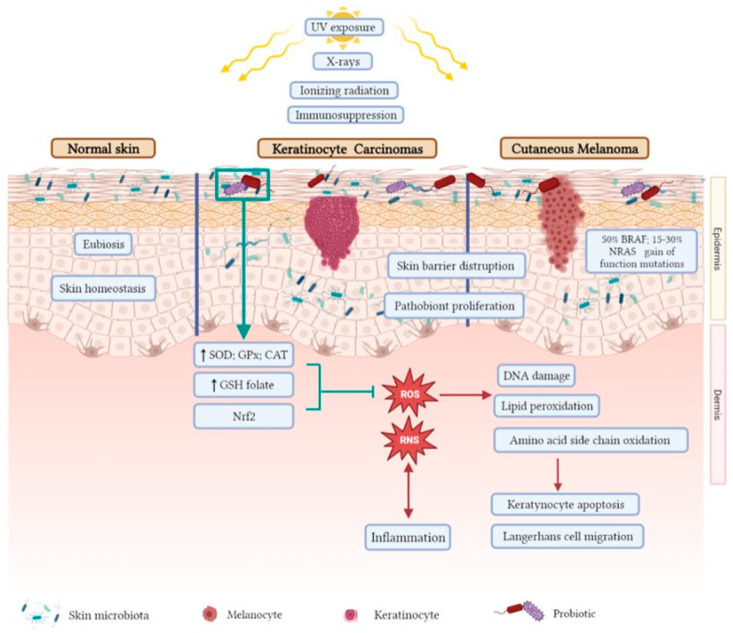
Keratinocyte carcinoma and cutaneous melanoma risk factors, microbiota status, and oxidative stress. This figure has been created with BioRender.

The role of the redox state in human tumors, including skin cancer (Figure 1), has been extensively reviewed elsewhere [109,157,158,159,160].

There is a general agreement in considering oxidative stress as one of the major forces in cancer initiation and antioxidants as one of the players with a protective role in the initial stages of this process. Indeed, accumulating evidence shows that enhanced ROS production triggers the occurrence and development of melanoma, SCC, and BCC through both genotoxic and non-genotoxic pathways [109]. UV exposure and other players of the skin exposome are the major causes of this ROS/RNS rise (see Section 4.1). Conversely, the induction of the antioxidant cellular response, sustained by the activation of Nrf2, protects against UVR genotoxicity [161,162], and the inhibition of Nrf2 increased susceptibility to carcinogens and accelerated the onset of skin cancer development [163].

However, numerous pieces of evidence point out that this theory is too simplistic. Indeed, the use of antioxidants as antitumor agents has not given the expected results. For instance, the administration of antioxidants, such as *N*-acetylcysteine (NAC) or the soluble vitamin E analogue Trolox, increased melanoma metastasis in a genetically engineered mouse model [164]; moreover, the intake of vitamin A or E supplement was associated with an increase in both BCC and SCC risk in women [165]. Thus, mounting evidence has recognized that both pro- and antioxidant species have a dual role as tumor-promoting and tumor-suppressing forces [109,166,167]. Indeed, ROS can induce skin carcinogenesis, but higher doses lead to cell death/apoptosis; the antioxidant Nrf2, traditionally considered a tumor suppressor for its cytoprotective functions, is activated during tumor progression, and its content is higher in chemo-resistant cells [168,169,170].

Redox adaptative homeostasis has been recognized as responsible for this apparent paradox: after the initial phases of carcinogenesis, the oxidative stress increase elicits the upregulation of the antioxidant species, contributing to cancer progression. Indeed, nuclear Nrf2 content correlated with nodular growth, invasive phenotype (Clark III–V), deeper Breslow depth thickness, and worse prognosis in melanoma patients [171]. The gain-of-function mutations of Nrf2 have been found to play a crucial role in the development of SCC, suggesting a possible oncogenic role [172]. SOD and GSH levels, but not Nrf2, were upregulated in BCC patients [173]. Nrf2 can potentiate radio-resistance in SCC cancer [174], and its epigenetic activation can decrease the sensitivity of esophageal SSC cells to cisplatin treatment [175]. Moreover, an increase in GSH and Nrf2 was also found in melanoma cells resistant to targeted therapy with MAPK inhibitors [176].

## 6. The Interplay between Microbiota, Oxidative Stress, and Skin Cancer

**Figure 2:**
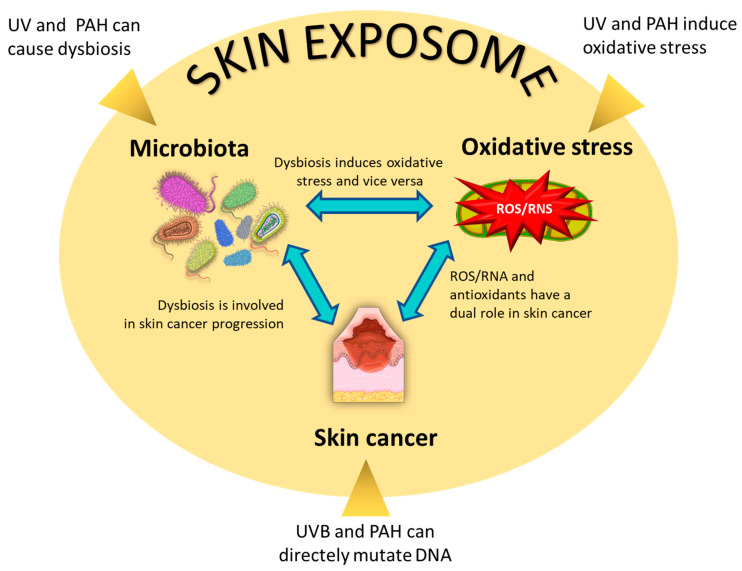
Interplay between microbiota, oxidative stress, and skin cancer, focusing on melanoma and keratinocyte cancers (basal and squamous cell carcinoma). Dysbiosis can induce oxidative stress and is involved in the onset and progression of skin cancer. ROS/RNS and antioxidants have a dual role in skin cancer; ROS/RNS induce skin carcinogenesis, and antioxidants, in these early phases, have a protective role; however, ROS/RNS high doses lead to cell death/apoptosis, and consequent redox adaptative homeostasis leads to the upregulation of the antioxidant species, contributing to cancer progression and chemo-resistance. Several components of the skin exposome can affect this interplay. For instance, the two environmental factors UV and PAH can cause dysbiosis, induce oxidative stress, and directly mutate DNA.

The possible interplay between microbiota, oxidative stress, and skin cancer (Figure 2) is suggested by a wide range of indirect evidence; however, the direct demonstration that dysbiosis affects cutaneous malignancy onset or its progression through the perturbation of the redox homeostasis is a largely unexplored area.

Indirect evidence has been provided with the demonstration that some bacterial species with antioxidant activity showed protection from the onset or progression of skin tumors. For example, the administration of *L. casei* YIT9018 or *L. reuteri*, two well-known probiotics with anti-inflammatory and antioxidant properties, reduced the incidence of melanoma, metastatic lesion, and significantly prolonged survival in B16 melanoma-bearing animals [177,178]. A diminished content of the skin commensal *C. acnes* and its secreted product RoxP, with antioxidant properties, has been found in BCC tissues [149]. Moreover, intra-tumoral injection of *C. acnes* in a melanoma mouse model significantly decreased melanoma growth and size [66].

Conversely, it has been also demonstrated that some microbiota species with pro-oxidant activities are associated with skin cancer progression. This is the case in *Malassezia* yeasts, in particular *M. furfur*, which has been found to contribute to BBC promotion. Indeed, it has been proposed that its secreted metabolites, such as indirubin, FICZ, or ICZ [142], could act as skin carcinogens through the activation of AhR [179], a well-known major player in ROS production [116,117]. HPV, involved in cutaneous carcinogenesis [39], is able to induce oxidative stress and DNA damage [180]. *F. nucleatum*, involved in melanoma disease progression [61], has been found to induce oxidative stress [181]. Other indirect evidence can be obtained from studies in which well-known chemical skin carcinogens can induce dysbiosis, with a consequent prevalence of microbial species that other authors have shown to be capable of inducing oxidative stress. For example, Leung and collaborators [182] demonstrated that skin exposition to PAH pollutant, a well-known class of chemical carcinogens [183]), caused skin dysbiosis, leading to a prevalence of *Staphylococcus* and *Malassezia*, two microbial species able to induce oxidative stress [141,184].

As previously reported [114], exposure to fine particulate matter (PM2.5) adversely affects the skin microbiota equilibrium and stimulates the aryl hydrocarbon receptor (AhR) to produce ROS. In turn, AhR induction may subsequently play a crucial role in skin carcinogenesis [185].

Chen et al. [107] suggest that skin microbiota, affected by environmental factors, is fundamental for maintaining a balance of skin oxidative stress levels, whereas skin microbiota dysbiosis causes skin alterations by triggering inflammatory responses through ROS accumulation. Several pieces of evidence indicate that chronic inflammation is one of the hallmarks of microenvironmental-agent-mediated skin cancers and contributes to their development [186].

The major limitation of this indirect evidence is the impossibility of confidently attributing a pathogenetic role of the redox homeostasis perturbation associated with dysbiosis as a determining force in inducing skin cancer. Indeed, it remains to be clarified whether dysbiosis-associated oxidative stress can be a cause or a consequence of the tumorigenic process. Few studies have addressed this point, investigating, at the same time, the contribution of oxidative stress and skin dysbiosis in cancer induction, and, more importantly, demonstrating that dysbiosis affects cutaneous tumor onset or progression through the perturbation of redox homeostasis. One of the few examples is represented by the experimental work proposed by Krueger and collaborators [141]. These authors investigated the role of *S. aureus*, a bacterium that increases in several KCs, such as BCC and SCC, and also in the premalignant AK [40,97]. They have demonstrated that *S. aureus* metabolites induce the expression of several SCC biomarkers in keratinocytes, such as kallikrein serine proteases and keratins, factors that promote inflammation and keratinocyte proliferation, migration, and invasion [187], multiple CXC chemokines involved in skin cancer progression [188], and MMPs that enhance angiogenesis and promote tumor invasion and metastasis [189]. Moreover, *S. aureus* caused an increase in intracellular ROS and nitrosative species, DNA double-strand breaks, quantified as phosphorylation of histone H2A.X, and 8-OHdG, a common product of oxidative DNA damage in human keratinocytes [141].

## 7. Conclusions

Oxidative stress has become an attractive and strategic therapeutic target to fight skin cancer [109,190]. At the same time, the modulation of the human gut and skin microbiota is now also an area of robust investigation in this pathological context [86,92]. Several ongoing clinical trials involve microbiota manipulation in skin cancer (Table 1). Most of the studies focus on enhancing melanoma immunotherapy through changes in microbiota composition via fecal microbiota transplantation (FMT) or administration of live biotherapeutic products (LBP). Of note, most of these LBP are a mixture of non-pathogenic, non-toxigenic commensal bacterial strains, likely all with antioxidant properties. Interestingly, MRx0518 (see NCT03934827 https://clinicaltrials.gov/, accessed on 28 December 2022) is an LBP consisting of a proprietary strain of a bacterium belonging to the *Enterococcus* species with well-known antioxidant activities [191].

The direct interplay between dysbiotic conditions and oxidative stress has been more than suggested; however, the relationship between human microbiota, oxidative stress, and skin cancer is still an unexplored area. A more complete understanding is clearly needed for the development of successful and lasting antitumoral treatments free from drug or immune checkpoint inhibitor resistance (Figure 2). Therefore, the research in this field should focus on (i) an investigation at the same time of the contribution of oxidative stress and dysbiosis in the progression of skin cancer; (ii) deepening the extreme complexity of this phenomenon, considering the dual role played by some of the protagonists; and (iii) the identification of the possible cause–effect relationships.

In the last years, probiotics have been deeply studied regarding their beneficial effect, especially their defensive role against oxidative stress, even if little is still known about the mechanisms at the basis of this. They can regulate the redox state through their diverse enzymes and pathways, but many questions have to be answered, especially if the in vivo discoveries obtained in animal models are fully transferrable to humans, since the clinical trials on this theme are still few. Therefore, to have a complete picture of probiotic antioxidant properties in the skin cancer field also, further in vitro and in vivo studies are needed.

## Figures and Tables

**Table 1:** Select ongoing clinical trials involving manipulation of microbiota in skin cancer.

Trial Number	Study Title	Trial Description	Phase/ Location
Fecal Microbiota Transplantation (FMT)			
NCT03353402	FMT in Metastatic Melanoma Pts Who Failed Immunotherapy	Pts with metastatic melanoma who responded to immunotherapy serve as the fecal implant donors in melanoma pts resistant to CPI. Primary outcomes: incidence of FMT-related AE and proper implant engraftment. Secondary outcomes: changes in composition and activity of the immune cell population.	Phase 1 Israel
NCT03341143	FMT in Melanoma Pts	The donors are pts with advanced melanoma who have been treated successfully with CPI. CPI-resistant melanoma pts received FMT and treatment with Pembrolizumab, a PD-1 inhibitor. Primary outcomes: ORR: number of pts with objective responses (PR, CR). Secondary outcomes: AE, PFS, OS, changes in immune cell composition and functionalities.	Phase 2 United States
NCT05251389	FMT to Convert Response to Immunotherapy	This is a randomized double-blind intervention phase Ib/IIa trial in CPI refractory metastatic melanoma pts receiving either FMT of a CPI responding or FMT from a CPI non-responding donor, in combination with CPI. After both types of FMT, melanoma pts will continue their anti-PD-1 treatment. Primary outcomes: efficacy, defined as CBR: SD, PR, CR. Secondary outcomes: AE, PFS, changes in the gut microbiome, metabolome, and immune cell populations.	Phase 1 Phase 2 Netherlands
NCT05286294	Microbiota Transplant to Cancer Patients Who Have Failed Immunotherapy Using Feces from Clinical Responders	FMT in CPI non-responding pts with melanoma or SCC. Primary outcomes: AE, ORR. Secondary outcomes: OS, PFS, DRR, implant engraftment estimation, evaluation of the effect of therapy on quality of life, fatigue, and pain.	Phase 2 Norway
Live Biotherapeutic Product (LBP)			
NCT05354102	A First-in-human (FIH) Combination Treatment Study With a Single Dose Level of BMC128	Melanoma pts receive a combination treatment of BMC128, a live bio-therapeutic product composed of 4 commensal bacterial strains, natural inhabitants of the human intestinal tract, in combination with anti-PD-1 Nivolumab. Primary outcomes: AE. Secondary outcomes: ORR, CBR: SD, PR, CR, DRR.	Phase 1 Israel
NCT04208958	Study of VE800 and Nivolumab in Patients With Selected Types of Advanced or Metastatic Cancer	Melanoma pts receive a combination treatment of VE800, a live bio-therapeutic product consisting of 11 distinct non-pathogenic, non-toxigenic, commensal bacterial strains, in combination with anti-PD-1 Nivolumab. Primary outcomes: AE, ORR. Secondary outcomes: DRR, CBR, PFS, OS, pharmacokinetics studies of VE800.	Phase 1 Phase 2 United States
NCT03934827	MRx0518 in Patients With Solid Tumors Waiting Surgical Removal of the Tumor	Melanoma pts amenable to surgical resection receive MRx0518, a proprietary strain of a bacterium belonging to *Enterococcus* species, which is found in the gastrointestinal tract of approx. 25% of humans and is predicted, from preclinical studies, to produce beneficial effects in humans. The first part of the study is devoted to measuring the safety and tolerability of MRx0518; in case of successful evaluation, the study will continue as a randomized double-blinded with a placebo. Primary outcomes: AE, general health assessment. Secondary outcomes: tumor markers, OS.	Phase 1 United Kingdom

Abbreviations: AE, Adverse Events; CBR, Clinical Benefit Range; CPI, Checkpoint Inhibitors; CR, Complete Response; DRR, Duration of Objective Response; FMT, Fecal Microbiota Transplantation; ORR, Objective Response Rate; OS, Overall Survival; PFS, Progression-free Survival; Pts, Patients; PR, Partial Response; SCC, Squamous Cell Carcinoma; SD, Stable Disease.
